# Damage to Olfactory Organs of Adult Zebrafish Induced by Diesel Particulate Matter

**DOI:** 10.3390/ijms23010407

**Published:** 2021-12-30

**Authors:** Su Jeong Song, Bongkyun Park, Kyuhyung Jo, Chan-Sik Kim

**Affiliations:** 1 Korean Medicine Convergence Research Division, Korea Institute of Oriental Medicine, Daejeon 34054, Korea; song.sj87@kiom.re.kr (S.J.S.); bkpark@kiom.re.kr (B.P.); jopd7414@kiom.re.kr (K.J.); 2 Korean Convergence Medical Science, University of Science and Technology (UST), Daejeon 34054, Korea

**Keywords:** diesel particulate matter, toxicity, olfactory dysfunction, cilia and epithelial damage, zebrafish

## Abstract

Particulate matter (PM) is an environmental hazard that is associated with various human health risks. The olfactory system is directly exposed to PM; therefore, the influence of PM exposure on olfactory function must be investigated. In this study, we propose a zebrafish olfactory model to evaluate the effects of exposure to diesel particulate matter (DPM), which was labeled Korean diesel particulate matter (KDP20). KDP20 comprises heavy metals and polycyclic aromatic hydrocarbons (PAHs). KDP20 exposed olfactory organs exhibited reduced cilia and damaged epithelium. Olfactory dysfunction was confirmed using an odor-mediated behavior test. Furthermore, the olfactory damage was analyzed using Alcian blue and anti-calretinin staining. KDP20 exposed olfactory organs exhibited histological damages, such as increased goblet cells, decreased cell density, and calretinin level. Quantitative real-time polymerase chain reaction (qRT-PCR) revealed that PAHs exposure related genes (*AHR2* and *CYP1A*) were upregulated. Reactive oxidation stress (ROS) (*CAT*) and inflammation (*IL-1B*) related genes were upregulated. Furthermore, olfactory sensory neuron (OSN) related genes (*OMP* and *S100*) were downregulated. In conclusion, KDP20 exposure induced dysfunction of the olfactory system. Additionally, the zebrafish olfactory system exhibited a regenerative capacity with recovery conditions. Thus, this model may be used in future investigating PM-related diseases.

## 1. Introduction

Particulate matter (PM) is an air pollutant comprising a mixture of solid particles and lipid droplets. It contains harmful chemicals such as polycyclic aromatic hydrocarbons (PAHs), heavy metals, carbon monoxides, and nitrogen oxides [1]. PM is classified according to size. PM_10_ has a diameter of 10 µm or less, PM_2.5_ has a diameter of 2.5 µm or less, PM_1_ has a diameter of 1 µm or less, and PM_0.1_ has a diameter of 0.1 µm or less. [2] PM_0.1_ is classified as ultrafine particulate matter (UPM), and its toxicity is of relatively great concern because it can easily infiltrate the human body [3]. Natural sources of PM include dust, volcanic ash, and ash and smoke from forest fires. PM is also found in smoke, fumes, factories, cooking processes, and garbage incineration [4]. The frequency and concentration of PMs has increased recently owing to an upsurge of industrialization and an increase in the use of fossil fuels [5]. In 2005, the World Health Organization (WHO) advised that the annual maximum acceptable average concentration of PM_10_ was below 20 µg/m^3^. However, in Korea, the annual average concentration of PM_10_ was 42 µg/m^3^ in 2019. Furthermore, the annual average concentration of PM_2.5_ in Bangladesh, Pakistan, and India was 77.1, 59.0, and 51.9 µg/m^3^, respectively, in 2020. These values exceeded the WHO recommendation of 10 µg/m^3^; therefore, the concentration of PM is high enough to affect health. This makes PM an environmental and health problem, and its effects on human health have been studied and reported [6].

The WHO has classified PM as a Group 1 carcinogen [7]. PM exposure induces reactive oxidative stress (ROS) [8,9], inflammation [10,11], mitochondrial dysfunction [12], and autophagy [13,14]. Additionally, PM exposure can exacerbate respiratory diseases such as asthma and bronchitis, as well as cardiovascular diseases such as myocardial infarction and vascular disruption [15,16]. One of the systems affected by PM exposure is the olfactory system. It is the most easily exposed system because PM directly enters the olfactory system through breathing, thus causing olfactory damage or worsening olfactory-related diseases. Therefore, recent studies on the association between PM exposure and olfactory dysfunction have garnered increasing interest. In urban areas of the United States, exposure to PM is associated with olfactory dysfunction, particularly among the 57–64 years age group [17]. One in vitro study demonstrated that PM from urban air induces mitochondrial dysfunction in human olfactory mucosal cells [18]. Urban air pollution damages olfactory neurons and bulbs among young adults and children [19]. As such, PM exposure has been reported to have detrimental effects on olfactory function. Therefore, an animal model that can be used to evaluate the damage of olfactory neurons or nerves caused by PM exposure is required but has not yet been established.

Zebrafish (*Danio rerio*) have been used as an animal model in the study of diseases and toxicity, as they are reported to have numerous advantages over other animal models [20]. For example, the upkeep of zebrafish is easier and cheaper than that of other animal models because of their small size, rapid development, and remarkable fertilization rate [21]. Zebrafish are also a remarkable genetic model because of their genetic similarity to mammals, including humans; moreover, the genetic modification of zebrafish is relatively easy. As vertebrates, the major organs and tissues of zebrafish—such as muscles, blood, intestines, kidneys, liver, and eyes—are similar to those in humans [22,23]. The embryos and larvae of zebrafish are transparent, making it possible to observe or track a fluorescent signal in real time. Therefore, zebrafish are useful for drug screening [24].

Zebrafish provide an ideal animal model for the study of the olfactory system. They have a well-developed sense of smell, which is associated with specific functions such as reproduction, feeding, and protection from predators [25]. The functional properties and anatomic components of the zebrafish olfactory system are similar to those of mammals [26]. The zebrafish olfactory system comprises a pair of peripheral olfactory organs and olfactory bulbs. The olfactory organs are connected to the olfactory bulbs, and together, they constitute the forebrain region. Olfactory organs of zebrafish have a sensory epithelium with olfactory sensory neurons (OSNs) to identify odors [27]. Therefore, zebrafish can be used as a representative animal model to study olfactory dysfunction. Zebrafish can regenerate their central nervous system (CNS) easily when it has been damaged, and this includes the olfactory system [27]. Olfactory damage can be tested using an odor-mediated behavior test [27]. Furthermore, zebrafish are highly sensitive to environmental pollutants, such as toxic heavy metals, endocrine disruptors, and PAHs [28]. Therefore, the olfactory systems of zebrafish larvae and adult zebrafish can be potentially used as bio-indicators for exposure to various pollutants [29]. These advantages make zebrafish an ideal olfactory model for studying regeneration, reorganization, and damage mechanisms following injury or toxic exposure.

In this study, we investigated DPM-induced damage of zebrafish olfactory organs and its functional deterioration. This study focused on the damage of OSNs distributed in the epidermis of olfactory organs directly affected by PM exposure. We analyzed markers of olfactory damage such as morphology of cilia and epithelium; loss of olfactory function; mucin and goblet cell damage; calretinin expression level; and change of mRNA level related to PAHs exposure, ROS, inflammation, and OSNs.

## 2. Results and Discussion

### 2.1. Physical Characterization of KDP20

**Figure 1:**
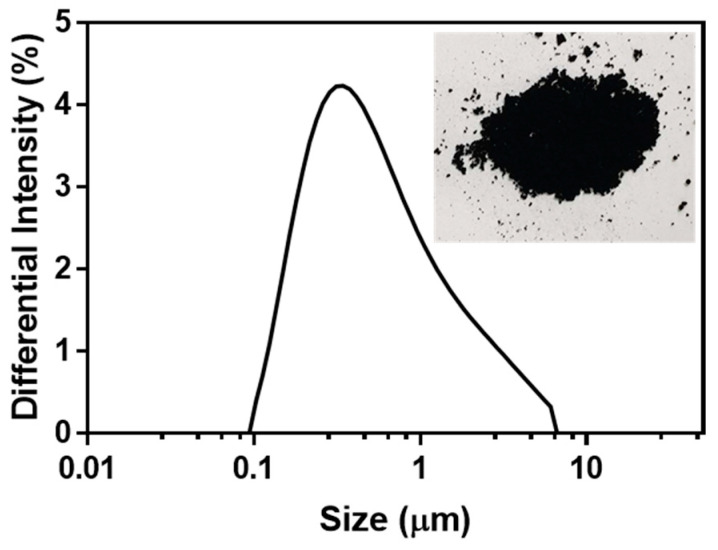
Graph of size distribution by dynamic light scattering (DLS) analysis and image of KDP20. The differential intensity distribution (%) of KDP20 is presented by the diameter size (µm, on a log scale). The size distribution was obtained through repeated measurements (60 times).

The KDP20 collected had the consistency of fine, black dust. It was analyzed for size distribution, elements, and PAH content to identify biotoxicity. Size distribution analysis showed that the average size was approximately 800 nm and the overall distribution was smaller than PM_10_, with approximately 70% of the distribution of KDP20 below PM_1_ (Figure 1). This small size of KDP20 likely results in easy permeation to directly exposed organs such as olfactory organs, eyes, and the skin, which can lead to high toxicity levels. In the PAH analysis using GC-MS, KDP20 contained phenanthrene, fluoranthene, and pyrene (Table 1). These PAHs have been reported to induce gene expression of the cytochrome P450 (CYP) enzyme group, which produces immune toxicity and oxidative metabolites [30,31]. Elemental analysis revealed that KDP20 comprised sulfur (S), iron (Fe), calcium (Ca), zinc (Zn), aluminum (Al), magnesium (Mg), sodium (Na), chlorine (Cl), potassium (K), copper (Cu), chromium (Cr), manganese (Mg), nickel (Ni), lead (Pb), titanium (Ti), and mercury (Hg) (presented in order of quantity) (Table 2). The S content was highest at 14,400 mg/kg, and was in the form of sulfur dioxide (SO_2_), generated by the combustion of fuel. Exposure to SO_2_ leads to the damage of mucosal layers and inflammation [32,33]. KDP20 contains various heavy metals such as Fe, Zn, Al, Cu, Mn, Ni, Pb, Ti, and Hg. These heavy metals are toxic, and can result in ROS generation, apoptosis, DNA damage, and enzyme inactivation. Among them, Cd, Pd, and Hg have been reported to have a high degree of toxicity and harmful effects on human health [34,35]. With respect to their effects on the zebrafish olfactory system, Cd exposure was reported to reduce the number of olfactory sensory neurons (OSNs) and increase ROS [36,37]. Cu exposure induces and decreases ciliated OSNs, olfactory receptors (ORs), and ionic channel transcripts [38]. Therefore, KDP20, which includes PAHs and harmful elements, is potentially toxic to the zebrafish olfactory system.

### 2.2. Survival Rate According to KDP20 Concentration

**Figure 2:**
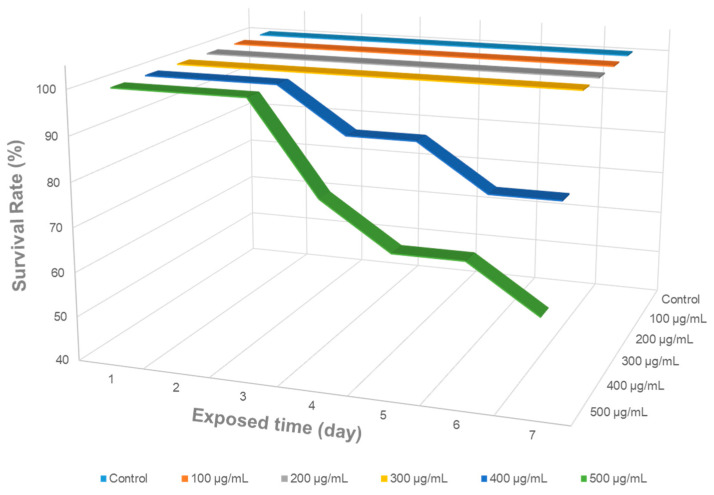
Survival rate (%) of control and zebrafish exposed to different concentrations of KDP20 (100, 200, 300, 400, and 500 µg/mL) for 1 to 7 days. The survival rates were recorded and observed daily (*n* = 10).

Prior to the study, zebrafish survival rate was analyzed after exposure to various concentrations of KDP20 (100, 200, 300, 400, and 500 µg/mL) for 7 days to determine the appropriate KDP20 exposure conditions to cause olfactory damage without affecting survival rate (Figure 2). Because the survival rates of control at 100, 200, and 300 µg/mL were equal, the readability was low; therefore, a three-dimensional graph was introduced and displayed. KDP20 in water floated due to buoyancy, and it was possible to effectively expose KDP20 to zebrafish. In the control group, all the zebrafish survived for 7 days. The groups exposed to 100, 200, and 300 µg/mL of KDP20 for 7 days exhibited a 100% survival rate. However, those exposed to 400 and 500 µg/mL of KDP20 exhibited a decreased survival rate after 4 days of incubation. In particular, the group exposed to 500 µg/mL of KDP20 exhibited a 50% survival rate after 7 days. These results show that KDP20, which contains several toxic substances such as PAHs and heavy metals, affected the survival rate upon prolonged exposure. Therefore, we introduced a 300 µg/mL environment that does not interfere with survival to confirm the effect of KDP20 exposure on zebrafish olfactory organs.

### 2.3. Field-Emission Scanning Electron Microscopy Analysis

**Figure 3:**
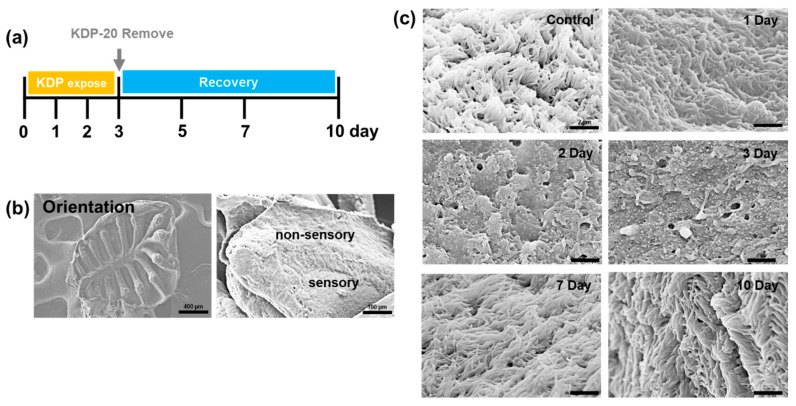
(**a**) Scheme of KDP20 exposure experiment. (**b**) Orientation of olfactory organs. Olfactory organ has rosette-like shape and contains non-sensory and sensory area. To analyze the damage of KDP20 exposure to olfactory sensory neurons (OSNs), we observed cilia and the epithelium of the sensory area. Scale bar = 100 µm. (**c**) Field emission scanning electron microscope (FE-SEM) images of cilia in the sensory area of control, damage group (1, 2, and 3 days), and recovery group (7 and 10 days). Scale bar = 2 µm.

The zebrafish were exposed to 300 µg/mL KDP20 for three days and allowed to recover in clean water for an additional 7 days (Figure 3a). The olfactory organ has an epithelium with several lamellae with a cup-shaped structure known as a rosette. Lamellae are composed of a sensory area located in the central region and a non-sensory area located dorsally. The non-sensory area has cilia that are considerably longer than the sensory epithelium. Therefore, the sensory area can easily be identified in the overview mode using FE-SEM (Figure 3b). The sensory epithelium has pear, microvillous, crypt, kappa, and ciliated OSNs, which are involved in odor-mediated behaviors such as foraging, mating, and socializing [27,39]. Therefore, sensory epithelium damage is an indicator of olfactory system impairment. We observed the epithelial surface in the sensory area using FE-SEM (Figure 3c). The control group exhibited a healthy epithelium with dense cilia. The 1 day group exhibited no significant damage, but the 2 day group exhibited an absence of multiple cilia. The three-day exposure group exhibited damage such as the reduction of cilia and changes in surface morphology. Olfactory damage similar to that observed in this study was also reported following exposure to Zn and Hg [40,41], and can be attributed to various harmful substances in KDP20. These results indicate the possibility of damage to OSNs owing to KDP20 exposure. Meanwhile, the recovery group presented a gradual regeneration of the cilia and improved epithelial morphology after 7 and 10 days. As the degree of recovery in both 7 and 10 days was similar, we used zebrafish accorded a 7 day recovery period in the histological analysis.

### 2.4. Elemental Analysis Using Scanning Electron Microscopy-Energy Dispersive X-ray Spectroscopy

**Figure 4:**
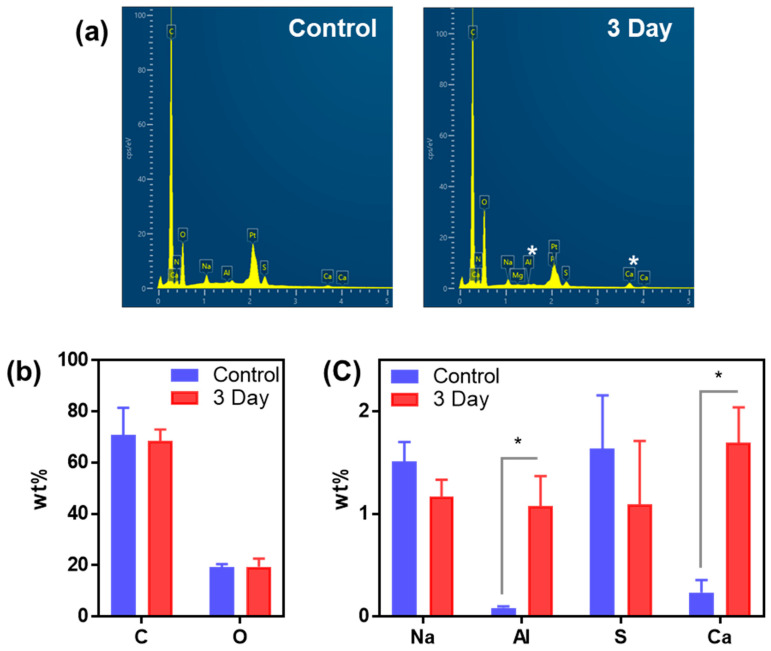
Elemental analysis in KDP20 three-day exposed olfactory organs using energy dispersive X-ray spectroscopy (EDS). (**a**) EDS spectrum of control and KDP20 three-day exposure groups; (**b**) Carbon (C) and oxygen (O); (**c**) sodium (Na), aluminum (Al), sulfur (S), and calcium (Ca) contents of control and KDP20 three-day exposure groups. Values are reported as mean ± SEM of three distinct olfactory organ samples. Statistical analysis by *t*-test, * *p* < 0.05.

In the FE-SEM analysis, damage to the sensory epithelium was observed after KDP20 exposure. Therefore, exposure to KDP20 can be assumed to have been sufficient and to have penetrated the olfactory system. Subsequently, we confirmed the residual component of KDP20 via SEM-EDS analysis. The three-day exposure group exhibited the most severe damage to the olfactory organs, especially in the sensory area, which was introduced for EDS analysis. The EDS spectra of the control and three-day (Figure 4a) exposure groups were compared, and toxic elements were observed with relatively high levels of C, O, Na, S, Al, and Ca (Figure 4b,c). The levels of C, O, Na, and S were similar, whereas Al and Ca levels were higher following the three-day exposure group than in the control group. Al and Ca were present in large quantities at 3530 and 5420 mg/kg, respectively. Therefore, they were expected to be deposited on the olfactory organs after KDP20 exposure. In particular, Al exposure has been reported to induce muco-ciliary clearance and mucosal layer damage [42], and it is related to the cilia defects and epithelial damage to the olfactory organs, as shown by FE-SEM analysis.

### 2.5. Odor-Mediated Behavior Test

**Figure 5:**
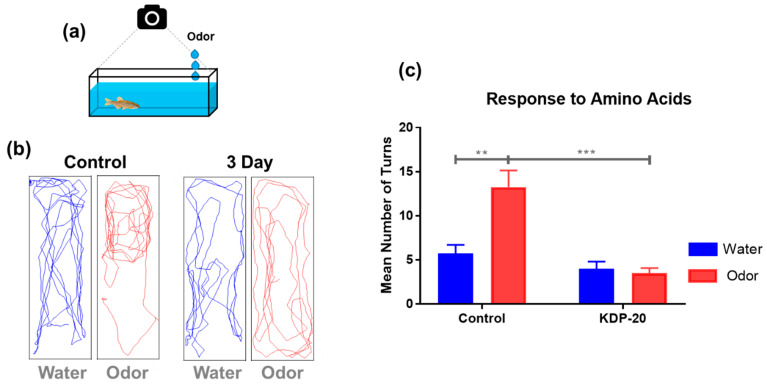
Odor-mediated behavior test of exposed zebrafish exposed to KDP20 for three days. (**a**) Scheme of odor-mediated behavior test. Odor is an amino acid mixture (alanine, cysteine, histidine, methionine, and valine; 10 mM each). The behavior of zebrafish was recorded aerially using a digital camera for 1 min per water and odor treatment. The recorded videos were analyzed using the animal tracker plug-in of Image J 1.52a software. (**b**) Behavior tracking image by water and odor treatment for one min. (**c**) Graph of mean number of turns in water and odor treatment for one min. Values are reported as mean ± SEM of five distinct olfactory samples. Statistical analysis by one-way ANOVA, ** *p* < 0.01, *** *p* < 0.001.

To investigate olfactory damage following KDP20 exposure, behavioral tests were performed using a mixture of water and amino acids (Figure 5a). Amino acids are food-related olfactory signals that elicit an attractive response. As a control, water treatment was administered for comparison. For this analysis, the three-day exposure group with severe damage was also selected. Prior to testing the behavioral response to the water and odor mixtures (amino acids), the zebrafish were stabilized in the testing tank. During stabilization, zebrafish exhibited typical behaviors of exploration and darting, as described by the zebrafish behavior catalog (ZBC) [43]. After the stabilization period, the zebrafish exhibited general swimming behaviors of moderate speed, intermittent turns, and the absence of rapid movements. The odor-mediated behavior tests were analyzed using movement tracking and quantification of the number of rapid turns. Movement tracking was performed for 1 min in a mixture of water and odor, which confirmed the overall response to odor exposure (Figure 5b). Zebrafish from the water treatment group exhibited general swimming behaviors in both the control and three-day exposure group. These results show that zebrafish do not respond to odor caused by water treatment, as with external physical factors. The odor treatment presented a significant difference between the control and the three-day exposure group. Zebrafish from the control group exhibited an active feeding response in odor treatment. They moved rapidly around the area, making sharp turns, indicating a normal feeding response. However, the three-day exposure group did not exhibit any behavior corresponding to the odor response. Quantification of the number of turns also showed a similar pattern to the behavior tracking analysis (Figure 5c). The odor-treated control group exhibited a higher number of turns than the water treatment group (*p* < 0.01), whereas the three-day exposure odor-treated group exhibited a notable difference to the water treatment group. In addition, the odor-treated control group exhibited a higher number of turns than the three-day exposure groups (*p* < 0.01). The odor-mediated behavior test demonstrated that KDP20 exposure induces a decrease in olfactory function in zebrafish adults.

### 2.6. Histopathology Analysis

**Figure 6:**
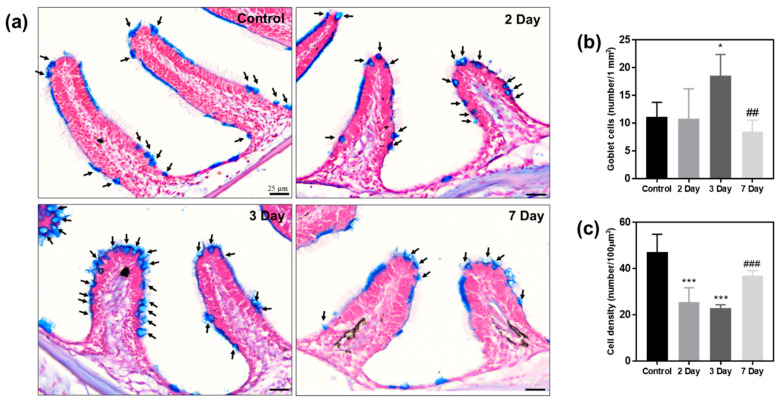
Histopathology analysis of zebrafish olfactory organs using Alcian blue and nuclear red fast red staining. (**a**) Images of control, damage group (2 and 3 days) and recovery group (7 days), scale bar = 25 µm. To analyze the damage to olfactory organs, the number of goblet cells and the cell density were quantified using Image J 1.52a software. (**b**) Number of goblet cell per 1 mm^2^. (**c**) Cell density analysis per 100 µm^2^. Values are reported as mean ± SEM of five distinct olfactory samples. Statistical analysis by one-way ANOVA, * *p* < 0.05, *** *p* < 0.001 versus control; ^##^ *p* < 0.01, ^###^ *p* < 0.001 versus three-days.

The histopathology of olfactory organs was analyzed using Alcian blue staining. Alcian blue and nuclear fast red staining are used to observe the mucosal layer and goblet cells, and can analyze overall tissue changes. Alcian blue binds to glycosaminoglycan and mucopolysaccharide, and can selectively stain mucin-secreting goblet cells and mucin layer. In addition, goblet cells can be identified using their distinctive goblet shape. Histological images are shown in Figure 6a, and the analysis focused on the number of goblet cells, and cell density.

Damage to the mucosal layer and goblet cells was analyzed, indicated by a blue signal after Alcian blue staining. On days 2 and 3 of exposure to KDP20, irregular mucosal layer and goblet cells that secreted mucus were observed and compared to the control group. These results show the presence of a protective response against damage caused by KDP20 exposure. On day 7 of recovery, the mucin layer and goblet cells were normal. Quantification of the goblet cells per 1 mm^2^ area showed that they had increased significantly in the zebrafish exposed to KDP20 for three days compared to the control (*p* < 0.05) and decreased significantly in the 7 day recovery group compared to the three-day recovery group (*p* < 0.01). The cell density per 100 µm^2^ was analyzed. The cell density was reduced after two and three days compared to that of the control (*p* < 0.001), and increased after 7 days of recovery compared to the values observed after exposure for 3 days (*p* < 0.001). The images also showed differences in the cell density for each condition. Histological analysis of goblet cells, mucin layer, and cell density confirmed that KDP20 exposure caused damage to the tissue and mucosal layer of zebrafish olfactory organs. In addition, recovery was confirmed to be possible in a clean environment without KDP20.

### 2.7. Immunohistochemistry of Anti-Calretinin

**Figure 7:**
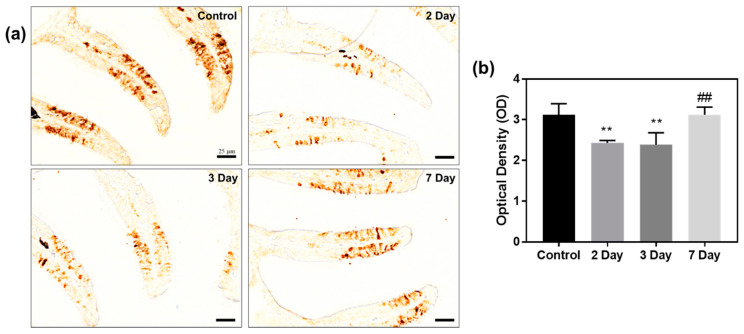
Immuno-histochemistry staining of anti-calretinin. (**a**) Images of anti-calretinin staining of control, damage group (2 and 3 days) and recovery group (7 days). Scale bar = 25 µm. (**b**) Graph of quantified calretinin intensity of optical density (OD) using image J 1.52a software. Values are reported as mean ± SEM of five distinct olfactory samples. Statistical analysis by one-way ANOVA, ** *p* < 0.01 versus control; ^##^ *p* < 0.01 versus three-days.

Because zebrafish exposed to KDP20 exhibited decreased olfactory function in behavioral experiments, immunohistochemistry using anti-calretinin was performed to analyze the mechanism underlying the damage observed. Calretinin is a calcium-binding protein found in neurons, which labels distinct groups of neurons in the peripheral and central nervous systems [44]. When calretinin is used to stain olfactory organs, it can be used as a biomarker for olfactory neurons in the epithelium. Therefore, anti-calretinin staining was performed to analyze the damage to OSNs following KDP20 exposure. Anti-calretinin staining was concentrated in the sensory areas (Figure 7a). In the two- and three-day groups, the intensity of calretinin decreased compared to that of the control group in the 7 day recovery period. The quantified data also significantly decreased in intensity on days 2 and 3 compared to the control (*p* < 0.01) and recovered significantly on day 7 of recovery group compared to 3 day (*p* < 0.01) (Figure 7b). Calretinin immunohistochemistry confirmed that KDP20 exposure damaged the tissue and mucosal layer of the olfactory organs, and the neurons responsible for olfactory function.

### 2.8. Real-Time Polymerase Chain Reaction Assay

**Figure 8:**
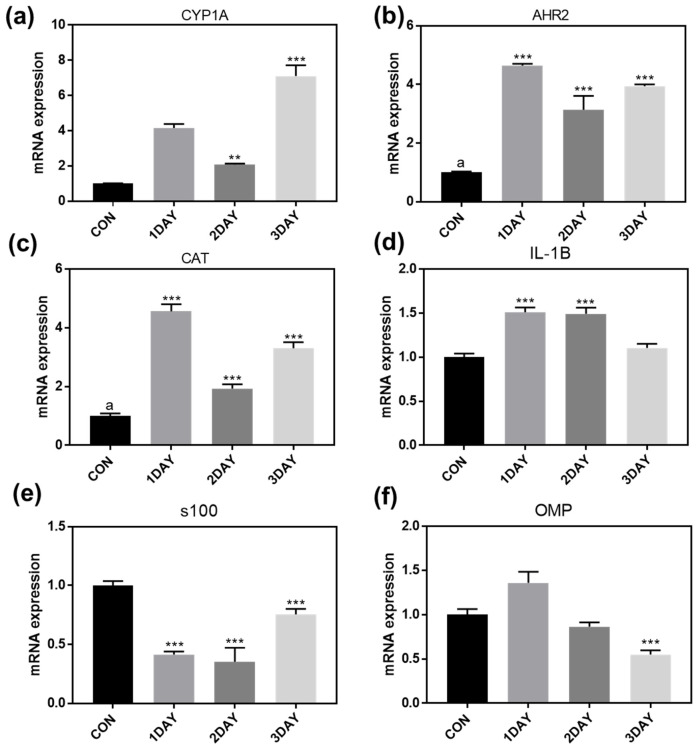
qRT-PCR analysis of olfactory organs of 1, 2, and 3 day KDP20 exposure. Gene expression was normalized relative to the expression of beta-actin. Expression of (**a**) *CYP1A*, (**b**) *AHR2*, (**c**) *CAT*, (**d**) *IL-1B*, (**e**) *S100*, and (**f**) *OMP*. Values are reported as mean ± SEM of four distinct olfactory samples. Statistical analysis by one-way ANOVA, ** *p* < 0.01, *** *p* < 0.001 versus control.

In previous analyses, we confirmed that KDP20 exposure can induce damage to the olfactory organs. To determine its effect on zebrafish gene expression, RT-PCR analysis was performed on days 1, 2, and 3 of KDP20 exposure. (Figure 8) RT-PCR analysis was performed for cytochrome p450 (*CYP1A* and *AHR2*), ROS (*CAT*), inflammation (*IL-1B*), and olfactory organ (*S100*, *OMP*)-related genes. Cytochrome p450 is a protein involved in the metabolism of drugs or environmentally harmful substances. Zebrafish have been reported to express cytochrome p450 and *AHR2* as biomarkers when exposed to environmental pollutants, including PAHs [45,46]. Therefore, we analyzed the expression of *CYP1A* and *AHR2* to determine the effect of KDP20 exposure. Expression of both *CYP1A* and *AHR2* increased at all the time points assessed. These results indicate that PAHs of KDP20 bind to the AHR2 of the olfactory epithelium, which induces the expression of *CYP1A*. Increased CYP1A has the potential to induce ROS generation and inflammation, resulting in widespread damage. Therefore, we quantified the *CAT* and *IL-1B* levels. Compared to the control group, both *CAT* and *IL-1B* expression were increased at all the time points assessed. These results indicate that KDP20 exposure induces ROS generation and inflammation in olfactory organs. Furthermore, *S100* protein has been observed in a subpopulation of sensory neurons, which play a role in calcium binding [47]. Therefore, the *S100* gene level can be a biomarker for olfactory damage and dysfunction. S100 expression levels were lower than controls for all three time points. These results demonstrate that the sensory neurons of the olfactory organs were damaged by KDP20 exposure, as indicated by the anti-caletinine staining performed in this study. *OMP* is an olfactory marker protein that is restricted to the sensory area in the adult olfactory epithelium [48]. *OMP* expression levels were observed to decrease as the incubation time increases. After KDP20 exposure for 3 days, *OMP* levels decreased significantly compared to those of the control group (*p* < 0.001). Therefore, RT-PCR assays demonstrated that KDP20 exposure caused damage to the sensory neurons and olfactory epithelium related to the CYP1A pathway, ROS, and inflammation.

## 3. Materials and Methods

### 3.1. Materials

Sea salt, tricaine, alanine, cysteine, histidine, methionine, and valine were purchased from Sigma Aldrich (Burlington, VT, USA). Phosphate-buffered saline (PBS) and distilled water (DW) were obtained from Welgene (Seoul, Korea). Ethanol and 4% paraformaldehyde were purchased from Merck (Darmstadt, Germany).

### 3.2. Zebrafish Maintenance

Zebrafish care and experimental procedures complied with institutional guidelines and were approved by local ethical boards. Adult zebrafish were stored in a circulating system under standard conditions with a 14 h light and 10 h dark cycle at 28 °C. The system water was purified using 5- and 1-µm micro depth and activated-carbon filters. The pH of the system water was maintained between 6.8 and 7.5. If pH is lower than 6.8, it is adjusted by adding coral sand. Zebrafish were fed with hatched artemia three times.

### 3.3. KDP20 Collection and Analysis

KDP20 was collected at an automobile exhaust inspection center (Daejeon, Korea) and stored in a sealed container with a desiccant. Physical properties: size distribution, element contents, and PAH contents, were quantified. To confirm the size distribution, KDP20 was dispersed at a concentration of 0.05 µg/µL in distilled water (DW), sonicated for 30 min, transferred to 1.5 mL polymethylmethacrylate cuvette, and measured using dynamic laser scattering (DLS) instrument of ELS-Z2 (Otsuka Electronics, Tokyo, Japan). The size distribution was illustrated through repeated measurements (60 times). Elemental and PAHs contents were analyzed at the Korea Polymer Testing and Research Institute (KOPTRI, Seoul, South Korea). Elemental contents were measured using inductively coupled plasma optical emission spectrometry (ICP-OES), cold vapor atomic absorption spectrometry (CV-AAS), and combustion ion chromatography (C-IC) at a detection limit of 0.1 mg/kg. PAHs contents were measured by gas chromatography mass spectrometry (GC-MS) at a detection limit of 0.2 mg/kg.

### 3.4. Survival Rate

The stock KDP20 solution was prepared at a concentration of 10 mg/mL in DW with 0.01% polysorbate 20 and sonicated for 30 min. To determine the appropriate KDP20 concentration, the survival rate with respect to KDP20 concentration and incubation time was analyzed. Zebrafish (12–14 weeks old, female, *n* = 10 per group) were exposed to 100, 200, 300, 400, and 500 µg/mL of KDP20 concentrations in system water with a total volume of 1.5 L for one to 7 days. The daily survival rates of the zebrafish were observed and recorded.

### 3.5. Exposure to KDP20

Zebrafish (12–13 weeks old, female, *n* = 5 per each group) were exposed to a KDP20 concentration of 300 µg/mL in system water with a total volume of 500 mL for up to 3 days. After three days of exposure, and to analyze the recovery of their olfactory systems, the zebrafish was incubated in clean system water for up to a maximum of additional 7 days. To analyze damage and recovery according to incubation time, a damage group (1, 2, and 3 days) and recovery group (7 and 10 days) were prepared. After KDP20 exposure or recovery, zebrafish were euthanized using ice and 0.14 mg/mL tricaine and the olfactory organs were carefully collected using fine tweezers and an objective microscope.

### 3.6. Field Emission-Scanning Electron Microscopy and Energy-Dispersive X-ray Spectroscopy Measurement

The olfactory organs collected from the control and three-day KDP20 exposure groups were fixed in 4% paraformaldehyde for 16 h at 4 °C, washed with PBS, and dehydrated using 20%, 40%, 60%, 80%, and 100% ethanol treatments for 20 min, sequentially. The prepared olfactory organs were dried for 1 h 25 °C, loaded carefully on a carbon tape attached to the sample chamber, coated with osmium for 15 s, and observed under an FE-SEM (Hitachi, Tokyo, Japan) using a 5–10 kV acceleration voltage.

### 3.7. Odor-Mediated Behavior Test

To identify olfactory system dysfunction, odor-mediated behavior tests were designed and performed following recent olfactory related research [41,49]. The odor solution was an amino acid mixture (alanine, cysteine, histidine, methionine, and valine; 10 mM each). The prepared zebrafish (*n* = 5 per group) from the control and three-day KDP20 exposure groups were individually transferred to a behavior test tank (5 × 20 × 3 cm) with a total volume of 100 mL clean system water and incubated for 1.5 h. After stabilization, the behavior test tank with each zebrafish was placed in an LED light box. First, 50 µL of water was added at the end of the tank, and the olfactory reaction was recorded for 1 min using a digital camera mounted on the top of the tank. Similarly, the odor solution was used to analyze the effect of the odor. Water was introduced as a mechanical control. The recorded videos were analyzed using the animal tracker plug-in of Image J 1.52a software (NIH, Bethesda, MD, USA), and the behavior trajectory of the odor response was visualized. In addition, the degree of odor responses was analyzed using the number of turns by the treatment of water and odor solutions.

### 3.8. Real-Time Polymerase Chain Reaction RT-PCR Analysis

To analyze the mechanism underlying the damage to the olfactory organs by KDP20 exposure, a real-time polymerase chain reaction (RT-PCR) was performed. The isolated olfactory organs (*n* = 3 per each group) of the control and three-day KDP20 exposure groups were washed with PBS and stored at −80 °C until they were analyzed. The prepared samples were combined using a tissue homogenizer (Krackeler, New York, NY, USA) with beads for 3 min under 1500 rpm. RNA from olfactory organ tissue was extracted and purified using an RNA extraction kit (Invitrogen, Waltham, MA, USA). The yield and purity of the RNA was confirmed using the ratio of absorbance at 260 and 280 nm using a NanoDrop spectrophotometer (Thermo Fisher, Waltham, MA, USA). One µg of isolated RNA was reverse transcribed using a SuperScript II kit (Bio-Rad, Hercules, CA, USA) for complementary DNA (cDNA) synthesis. The prepared cDNA was subjected to RT-PCR with primers (Table 3) using an RT-PCR system (Applied Biosystems, Waltham, MA, USA). Gene expression of *CYP1A*, *AHR2*, *CAT*, *IL-1B*, *S100*, and *OMP* was normalized relative to the expression of beta-actin.

### 3.9. Histopathology and Immunohistochemistry

To determine the olfactory damage and recovery following KDP20 exposure, histopathological analysis, and immunohistochemistry (*n* = 5 per each group) were performed according to previously reported protocols [50]. Alcian blue and nuclear fast red (Vector Laboratories. lnc, San Francisco, CA, USA) staining were used to observe the mucin layer, goblet cells, and density. The number of goblet cells per one mm^2^ area, and cell density by number per 100 µm^2^ were quantified in the captured images using Image J 1.52a software. To confirm the expression levels of calretinin, anti-calretinin (Santa Cruz Biotechnology, Dallas, TX, USA) was used. The prepared slides were labeled using an LSAB kit (DAKO, Carpinteria, CA, USA) and observed using a DAB substrate kit (DAKO). The slide images were prepared using a slide scanner (3DHistech, Budapest, Hungary). The optical density of calretinin staining was calculated using Image J 1.52a software.

### 3.10. Data Analysis

Results are presented as mean ± SEM of multiple experiments. Significant differences between groups were analyzed using *t*-test, one-way analysis of variance (ANOVA), and Tukey’s multiple comparison using Prism 7.0 software (GraphPad, La Jolla, CA, USA). The graph showing the survival rate (Figure 2) was created using Microsoft Excel (Microsoft, Redmond, DC, USA).

## 4. Conclusions

KDP20 is a diesel particulate matter that is produced from the exhaust system of automobiles. Exposure to KDP20 is likely, especially among people living in urban areas. Therefore, the study of the health risks of diesel particulate matter, such as KDP20, is necessary. The olfactory organ is exposed to PM first, thereby necessitating a study of the mechanism of the damage caused.

In this study, we observed zebrafish olfactory damage by exposure to diesel particulate matter (DPM). The DPM was collected in Korea in 2020 and named Korea diesel particulate matter (KDP20). In addition, KDP20 contains harmful substances such as PAHs, metallic metals, and heavy metals; therefore, research on the toxicity of the olfactory system is important. The olfactory system of zebrafish exposed to KDP20 displayed damage and dysfunction.

Zebrafish adults were exposed to KDP20 for 3 days and then allowed to recover for 7 days in clean water. In the aquatic environment, KDP20 was suspended, and zebrafish were effectively exposed. Damage and recovery of the sensory area of the olfactory organ was confirmed by observing cilia using FE-SEM. FE-SEM analysis showed reduced cilia and epithelial damage in the sensory area after incubation time for recovery. Histological assays of Alcian blue revealed damage to the mucosal layer and tissue. Anti-calretinin staining revealed decreased calretinin level in the sensory area. Behavioral tests were performed following zebrafish exposure to KDP20 for 3 days, as the findings illustrated the dysfunction of the olfactory system caused by KDP20 exposure. To analyze the mechanism underlying damage induced by KDP20 exposure, RT-PCR was performed. *CYP1A*, *AHR2*, *CAT*, and *IL-1B* expression were upregulated and *S100* and *OMP* were decreased. KDP20 exposure induces damage and dysfunction in the olfactory system of adult zebrafish. In addition, when placed in a recovery tank, the zebrafish exhibited olfactory organ recovery, observed through histological and cilia examination.

PM is a pollutant that is dispersed in the air. Although this is a study on the harmful effect of PM on zebrafish under an aquatic environment, KDP20 exposure was confirmed to damage OSNs and increase the olfactory dysfunction. Therefore, our study can be used as basic research on the olfactory dysfunction caused by PM or exposure to environmental pollution. Future studies should be conducted to determine the mechanisms of the damage. In conclusion, this study identified a zebrafish model that can aid in the determination of the mechanism underlying damage caused by exposure to diesel particulate matter.

## Figures and Tables

**Table 1:** Polycyclic aromatic hydrocarbons (PAHs) contents of KDP20 by gas chromatography mass spectrometry (GC-MS) analysis at a detection limit of 0.2 mg/kg.

PAHs	Content (mg/kg)
Phenanthrene	42.0
Fluoranthene	40.6
Pyrene	2.5

**Table 2:** Elemental contents of KDP20 by coupled plasma optical emission spectrometry (ICP-OES), cold vapor atomic absorption spectrometry (CV-AAS), and combustion ion chromatography (C-IC) analysis at a detection limit of 0.1 mg/kg.

Element	Content (mg/kg)
S (Sulfur)	14,400
Fe (Iron)	14,100
Ca (Calcium)	5420
Zn (Zinc)	5180
Al (Aluminum)	3530
Mg (Magnesium)	673
Na (Sodium)	547
Cl (Chlorine)	511
K (Potassium)	455
Cu (Copper)	315
Cr (Chromium)	121
Mn (Manganese)	117
Ni (Nickel)	88.8
Pb (Lead)	62.2
Ti (Titanium)	58.7
Hg (Mercury)	0.024

**Table 3:** List of primers for qRT-PCR. Sequences are given from 5′ to 3′. Normalized gene (beta-actin). PAHs exposure related gene (*AHR2* and *CYP1A*). Oxidation related gene (*CAT*). Olfactory sensory neurons related gene (*OMP* and *S100*).

Gene	Primer Sequence	
Beta-actin	Forward	cccagacatcagggagtgat
	Reverse	cacaataccgtgctcaatgg
CYP1A	Forward	gacaggcgctcctaaaacag
	Reverse	ctgaacgccagactctttcc
AHR2	Forward	gcctgggataaaggaggaag
	Reverse	cagctccatcctgtccaaat
CAT	Forward	agtgctcctgacgtccagcca
	Reverse	tgaagaacgtgcgcacctggg
IL-1B	Forward	gctggagatccaaacggata
	Reverse	atacgcggtgctgataaacc
S100	Forward	gcagtgaaggagaca
	Reverse	atagagcattacgggtat
OMP	Forward	ggctctcttctggtca
	Reverse	ttgcgttataactccctt

## Data Availability

The data used to support the findings of the present study are available from the corresponding author upon request.
